# Formation of ZnO/Zn_0.5_Cd_0.5_Se Alloy Quantum Dots in the Presence of High Oleylamine Contents

**DOI:** 10.3390/nano9070999

**Published:** 2019-07-11

**Authors:** Yi-An Chen, Kuo-Hsien Chou, Yi-Yang Kuo, Cheng-Ye Wu, Po-Wen Hsiao, Po-Wei Chen, Shuo-Huang Yuan, Dong-Sing Wuu

**Affiliations:** 1 Department of Materials Science and Engineering, National Chung Hsing University, 145 Xingda Road, Taichung 40227, Taiwan; 2 Innovation and Development Center of Sustainable Agriculture, National Chung Hsing University, Taichung 40227, Taiwan; 3 Research Center for Sustainable Energy and Nanotechnology, National Chung Hsing University, Taichung 40227, Taiwan

**Keywords:** quantum dots, ZnO, one-pot method, Zn_0.5_Cd_0.5_Se, oleylamine

## Abstract

To the best of our knowledge, this report presents, for the first time, the schematic of the possible chemical reaction for a one-pot synthesis of Zn_0.5_Cd_0.5_Se alloy quantum dots (QDs) in the presence of low/high oleylamine (OLA) contents. For high OLA contents, high-resolution transmission electron microscopy (HRTEM) results showed that the average size of Zn_0.5_Cd_0.5_Se increases significantly from 4 to 9 nm with an increasing OLA content from 4 to 10 mL. First, [Zn(OAc)_2_]–OLA complex can be formed by a reaction between Zn(OAc)_2_ and OLA. Then, Fourier transform infrared (FTIR) spectroscopy and X-ray diffraction (XRD) data confirmed that ZnO is formed by thermal decomposition of the [Zn(OAc)_2_]–OLA complex. The results indicated that ZnO grew on the Zn_0.5_Cd_0.5_Se surface, thus increasing the particle size. For low OLA contents, HRTEM images were used to estimate the average sizes of the Zn_0.5_Cd_0.5_Se alloy QDs, which were approximately 8, 6, and 4 nm with OLA loadings of 0, 2, and 4 mL, respectively. We found that Zn(OAc)_2_ and OLA could form a [Zn(OAc)_2_]–OLA complex, which inhibited the growth of the Zn_0.5_Cd_0.5_Se alloy QDs, due to the decreasing reaction between Zn(oleic acid)_2_ and Se^2−^, which led to a decrease in particle size.

## 1. Introduction

The preparation and identification of quantum dots (QDs) have been widely studied, thus creating a new field of research. Different chemical methods have been reported for preparing QDs, such as microwave irradiation [1], solvothermal [2], and nonorganometallic precursor [3,4,5] methods, as well as the pyrolysis of single-molecular organometallic compounds [6,7], the organometallic precursor method [8], and the sonochemical method [9]. Preparing multicomponent alloy QDs involves more complicated steps than preparing single-component QDs. Thus, the fabrication of alloy QDs using the one-pot method has become a popular research topic since the late 2000s [10,11,12,13,14,15]. QDs have been used in a wide variety of applications such as light-emitting diodes, lasers [16,17], solar cells [18,19,20], photonic band-gap crystals [21], and biomedical labels [22,23,24]. QDs are quasi-zero-dimensional nanomaterials composed of typical inorganic semiconductors [25]. Because QDs have various emission wavelengths, they have attracted considerable attention in fields requiring special material, physical, and chemical properties [14,26]. The various emission wavelengths of multicomponent alloy QDs are the most widely investigated, given that many reaction parameters can be controlled. Although the emission wavelength of QDs can change with nanoparticle size, it can also be controlled through the ratio and composition of the precursors [14].

More recently, various ligands, such as trioctylphosphine (TOP) ligands, oleic acid (OA) ligands, and oleylamine (OLA) ligands, have been extensively investigated in order to determine the impact of size and ligand chemistry on the optical properties and growth of QDs [27,28]. Most researchers have focused on changing the emission wavelength of QDs by controlling the reaction time, temperature, and ratio and composition of precursors [27,28,29,30]. The synthesis of ternary Zn_x_Cd_1−x_Se alloy QDs has become well-establishment using the one-pot method. Various shell/core QD systems have been synthesized and reported, i.e., ZnS/Zn_x_Cd_1−x_Se, CdZnS/Zn_x_Cd_1−x_Se, and CdSe/Zn_x_Cd_1−x_Se, which are the most studied with simple synthetic control resulting in desired optical properties [30,31,32,33,34]. Previous studies have mostly used a two-step chemical reaction method to synthesize the core/shell ZnO/CdSe system and investigated emission wavelength, morphology, and growth evolution [35,36,37,38]. However, to the best of our knowledge, there are few reports on the core/shell ZnO/Zn_x_Cd_1−x_Se alloy QDs (not the ZnO/Zn_x_Cd_1−x_Se nanocables and hollow spheres) in the literature [19,39]. The effect of varying OLA contents on the growth evolution of ZnO/Zn_x_Cd_1−x_Se alloy QDs using a one-pot method has not yet been discussed. Thus, the growth evolution of ZnO/Zn_x_Cd_1−x_Se alloy QDs with various OLA contents is an important subject.

In the present study, we used a one-pot method to synthesize ternary Zn_0.5_Cd_0.5_Se alloy QDs. We investigated the effect of the OLA ligands on the emission wavelength, morphology, and growth evolution of these QDs, which were characterized along with their structure using high-resolution transmission electron microscopy (HRTEM), photoluminescence (PL), Fourier transform infrared (FTIR) spectroscopy, ultraviolet-visible (UV-vis) spectroscopy, X-ray photoelectron spectroscopy (XPS), and X-ray diffraction (XRD).

## 2. Materials and Methods

### 2.1. Materials

Zinc acetate (Zn(OAc)_2_, 99.99%), cadmium oxide (CdO, 99.99%), Se (99.99%), trioctylphosphine (TOP, 90%), 1-octadecene (ODE, 90%), OA (90%), OLA (80–90%), ethanol, and anhydrous toluene were purchased from Sigma-Aldrich (Uni-onward Trade Co., Ltd., New Taipei City, Taiwan) and used without any further purification.

### 2.2. Synthesis of Zn_0.5_Cd_0.5_Se Alloy QDs

Three different Zn_x_Cd_1−x_Se alloy QDs were prepared using a one-pot method. The feed Zn(OAc)_2_/CdO molar ratios were 0.5:0.5; the resulting products are hereinafter referred to as Zn_0.5_Cd_0.5_Se. To synthesize Zn_0.5_Cd_0.5_Se alloy QDs, 0.5 mmol of Zn(OAc)_2_, 0.5 mmol of CdO, and 5 mL of OA were placed in a four-neck flask along with different amounts of OLA (0, 2, 4, 6, or 10 mL) and ODE (10, 8, 6, 4, or 0 mL). Then, the mixture was heated to 150 °C under flowing high-purity Ar gas for 30 min. After 30 min, all the solids in the flask were completely dissolved, yielding a clear solution of Zn(OA)_2_ and Cd(OA)_2_. The solution was then heated to 300 °C while quickly injecting 4 mmol of Se in 4 mL of TOP into the four-neck flask. The reaction temperature was maintained at 280 °C for 30 min to grow the Zn_0.5_Cd_0.5_Se alloy QDs. This solution was then rapidly added to ice toluene to terminate the reaction. The mixed solution was precipitated in excess ethanol and centrifuged at 5000 rpm for 10 min to separate the Zn_0.5_Cd_0.5_Se alloy QDs and the supernatant liquid phase was decanted to remove the excess reagent. Subsequently, purified Zn_0.5_Cd_0.5_Se alloy QDs in a nonpolar toluene solution were re-dispersed.

The alloy QDs are labeled Zn_0.5_Cd_0.5_Se-y, where y is the OLA content (mL) added during the synthesis process.

### 2.3. HRTEM

The morphology and size of all QDs were investigated using HRTEM (JEM-2100F, JEOL Ltd., Tokyo, Japan) with an accelerating voltage of 200 kV.

### 2.4. XRD

To determine the crystal structure of the QDs, XRD patterns were recorded using a Bruker D8 Advance diffractometer (Bruker AXS, Inc., Madison, WI, USA) over scanning ranges from 2θ = 20° to 70° at a scanning rate of 2°/min.

### 2.5. PL

The PL spectra were collected using a Hitachi F-2700 (Hitachi Ltd., Tokyo, Japan) fluorescence (excitation wavelength, λ_ex_ = 365 nm).

### 2.6. FTIR

The FTIR spectra were obtained on a Perkin-Elmer spectrometer (Waltham, MA, USA). One spectrum in the transmission mode from 400 to 4000 cm^−1^ was obtained after 20 scans at a 4 cm^−1^ resolution using the standard KBr disk method.

### 2.7. XPS

The chemical states and constituent compositions of the all samples were analyzed by XPS (ULVAC-PHI PHI 5000 Versa Probe, Kanagawa, Japan).

### 2.8. UV-vis Spectrophotometer

UV-vis spectra were performed on a Hitachi U-3010 (Hitachi Ltd., Tokyo, Japan) with a bandwidth 0.1 nm and a scanning speed at 200 nm/min.

## 3. Results and Discussion

### 3.1. Effect of Low OLA Content

**Figure 1:**
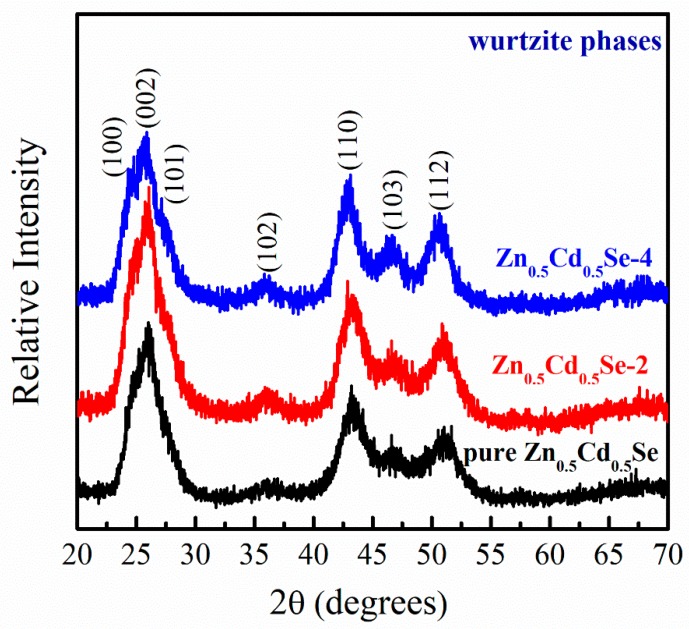
X-ray diffraction (XRD) patterns of Zn_0.5_Cd_0.5_Se alloy quantum dots (QDs) with low oleylamine (OLA) content.

According to the literature, both zinc-blende and wurtzite phases might form during the fabrication of ternary Zn_0.5_Cd_0.5_Se alloy QDs [31,32,33]. We studied the effect of low OLA content on the preparation of Zn_0.5_Cd_0.5_Se alloy QDs, where the initial OLA content was set to 0, 2, and 4 mL, and all other reaction parameters were fixed. All data were analyzed under the same parameters. Figure 1 shows the XRD patterns of Zn_0.5_Cd_0.5_Se alloy QDs prepared with various OLA contents. The patterns of pure Zn_0.5_Cd_0.5_Se alloy QDs clearly exhibited diffraction peaks at 2θ = 25.32° (100), 26.63° (002), 28.29° (101), 37.24° (102), 44.78° (110), 48.33° (103), and 53.07° (112), indicating a wurtzite crystal structure [31,34]. Because the wurtzite phase is thermodynamically more stable than the zinc-blende phase [34], these ternary pure Zn_0.5_Cd_0.5_Se alloy QDs predominantly developed a wurtzite structure. Thus, the Zn_0.5_Cd_0.5_Se alloy QDs with various OLA contents all exhibited a wurtzite crystal structure [31,34]. The crystal structures of Zn_0.5_Cd_0.5_Se-2 and Zn_0.5_Cd_0.5_Se-4 alloy QDs are the same as that of pure Zn_0.5_Cd_0.5_Se alloy QDs. These results suggest that adding OLA during synthesis does not change the crystal structure of Zn_0.5_Cd_0.5_Se alloy QDs.

**Figure 2:**
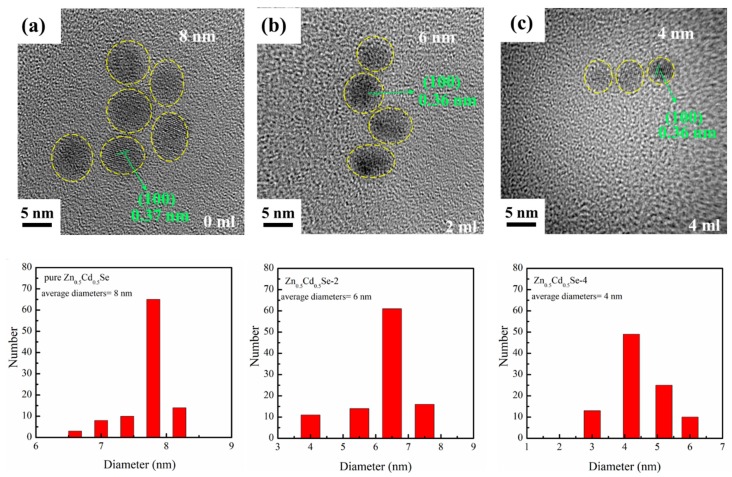
High-resolution transmission electron microscopy (HRTEM) images and size distributions showing the influence of low OLA content on (**a**) pure Zn_0.5_Cd_0.5_Se, (**b**) Zn_0.5_Cd_0.5_Se-2, and (**c**) Zn_0.5_Cd_0.5_Se-4 alloy QDs.

Figure 2 shows the HRTEM images and size distributions of various OLA contents on the preparation of Zn_0.5_Cd_0.5_Se alloy QDs. In these images, all alloy QDs are clearly monodispersed and quasi-spherical. The average diameters of the Zn_0.5_Cd_0.5_Se alloy QDs are estimated to be approximately 8, 6, and 4 nm for 0, 2, and 4 mL OLA loadings, respectively. Thus, the average diameters of the Zn_0.5_Cd_0.5_Se alloy QDs decreases as the OLA content increases in the reaction solution up to 4 mL. This decrease might be due to OLA inhibiting the growth of Zn_0.5_Cd_0.5_Se alloy QDs, thus decreasing the particle size (discussed in Section 3.2). In addition, the interplanar distances are estimated to be 0.37, 0.36, and 0.36 nm for pure Zn_0.5_Cd_0.5_Se, Zn_0.5_Cd_0.5_Se-2, and Zn_0.5_Cd_0.5_Se-4, respectively. This result suggests that adding OLA during synthesis does not change the crystal structure of Zn_0.5_Cd_0.5_Se alloy QDs and consistent with the XRD results.

**Figure 3:**
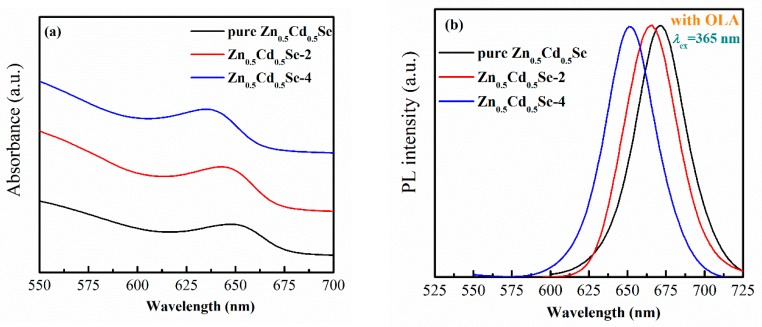
Influence of the low OLA content on the (**a**) UV-vis absorption spectra and (**b**) photoluminescence (PL) spectra of Zn_0.5_Cd_0.5_Se alloy QDs.

To understand the effect of adding OLA on the absorption and emission wavelength of Zn_0.5_Cd_0.5_Se alloy QDs, UV-vis absorption and PL spectra were employed. The UV-vis absorption and PL spectra of Zn_0.5_Cd_0.5_Se alloy QDs prepared with various OLA contents are shown in Figure 3. When the OLA content increased from 0 to 4 mL, the emission wavelength gradually blue-shifted from 671 to 651 nm, respectively. We observed that the first absorption feature blue-shifted from 649 to 635 nm. Thus, the absorption and emission wavelength of the Zn_0.5_Cd_0.5_Se alloy QDs blue-shifted with decreasing particle size, which is the opposite trend as that of Cd_3_P_2_ QDs [29]. Specifically, Miao et al. reported that the emission peaks of Cd_3_P_2_ QDs red-shifted with increasing OLA content [29], which implied that adding OLA is helpful to the growth of Cd_3_P_2_ QDs [29].

### 3.2. Effect of High OLA Content

**Figure 4:**
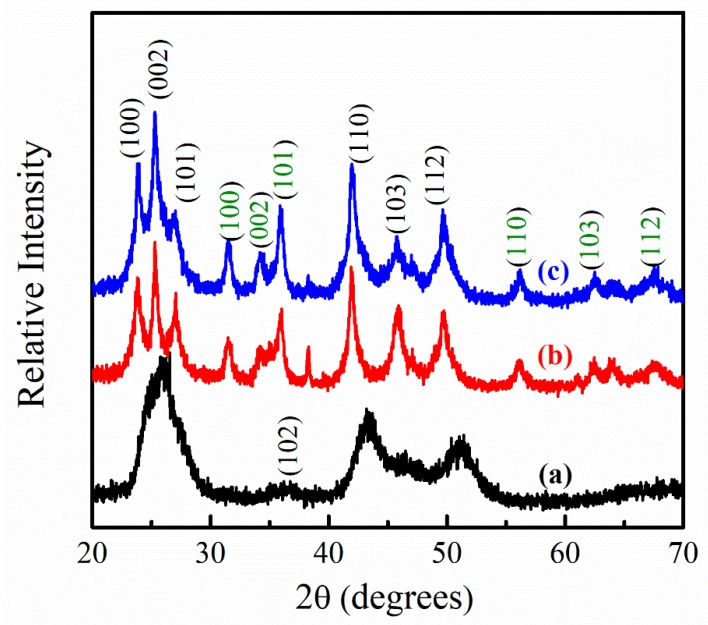
Effect of the high OLA content on the XRD patterns of (**a**) pure Zn_0.5_Cd_0.5_Se, (**b**) Zn_0.5_Cd_0.5_Se-6, and (**c**) Zn_0.5_Cd_0.5_Se-10 alloy QDs.

To investigate the crystal structure of pure Zn_0.5_Cd_0.5_Se alloy QDs with high OLA contents, we recorded XRD patterns, which are depicted in Figure 4. The pure Zn_0.5_Cd_0.5_Se alloy QDs exhibited a wurtzite crystal structure [31,34] and diffraction peaks at 2θ = 25.32°, 26.63°, 28.29°, 37.24°, 44.78°, 48.33°, and 53.07° were assigned to the (100), (002), (101), (102), (110), (103), and (112) planes, respectively. The XRD patterns of Zn_0.5_Cd_0.5_Se-6 and Zn_0.5_Cd_0.5_Se-10 (Figure 4) containing six extra diffraction peaks were different from that of pure Zn_0.5_Cd_0.5_Se. Six diffraction peaks appeared at 2θ values of 31.80°, 34.41°, 36.21°, 56.53°, 62.74°, and 67.80°, which correspond to the (100), (002), (101), (110), (103), and (112) planes of the wurtzite ZnO structure (JCPDS card no. 36-1451), respectively [40]. Since both Zn_0.5_Cd_0.5_Se and ZnO have a wurtzite crystal structure, they provided good lattice matching. Thus, we conjectured that the wurtzite ZnO structure might preferentially grow on the Zn_0.5_Cd_0.5_Se surface with high OLA contents.

**Figure 5:**
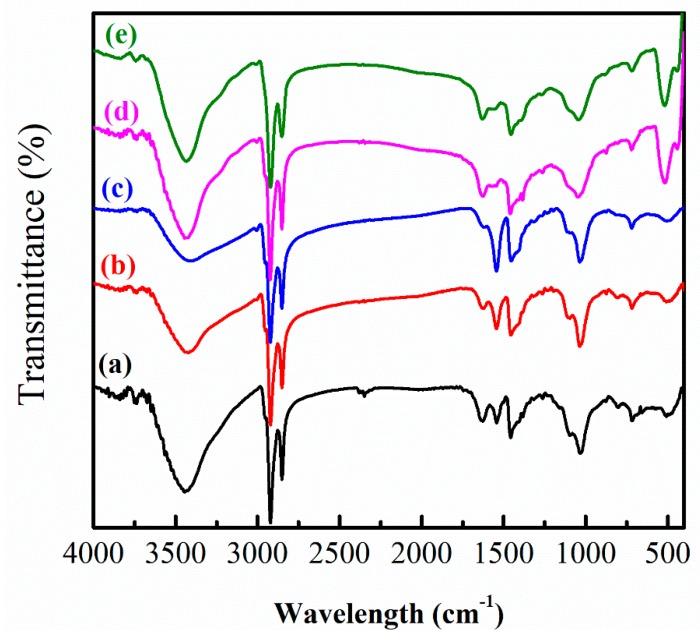
FTIR spectra showing the influence of the various OLA content on (**a**) pure Zn_0.5_Cd_0.5_Se, (**b**) Zn_0.5_Cd_0.5_Se-2, (**c**) Zn_0.5_Cd_0.5_Se-4, (**d**) Zn_0.5_Cd_0.5_Se-6, and (**e**) Zn_0.5_Cd_0.5_Se-10 alloy QDs.

To understand the XRD results, FTIR was used to explain the differences between Zn_0.5_Cd_0.5_Se and Zn_0.5_Cd_0.5_Se-y. Figure 5 presents the FTIR spectra of Zn_0.5_Cd_0.5_Se and Zn_0.5_Cd_0.5_Se-y. For all samples, the FTIR data revealed strong absorption peaks at 2986–3686 cm^−1^, which were assigned to the carboxylic acid OH stretching mode of OA and N–H stretching vibration of OLA. The strong absorption peaks at 2853–3005 cm^−1^ were attributed to the =C–H and C–H stretching vibration of ligands (OA and/or OLA). The absorption peaks of the ligands (OA and/or OLA) are located in the wavenumber region from 630 to 1750 cm^−1^. However, compared to the FTIR spectra of Zn_0.5_Cd_0.5_Se, Zn_0.5_Cd_0.5_Se-2 and Zn_0.5_Cd_0.5_Se-4, the FTIR spectra of Zn_0.5_Cd_0.5_Se-6 and Zn_0.5_Cd_0.5_Se-10 contained two extra absorption peaks at 530 and 435 cm^−1^. The absorption peaks associated with Zn‒O stretching band clearly appeared at 530 and 435 cm^−1^, confirming the formation of ZnO [41]. These results demonstrate that this chemical reaction could produce ZnO when with high OLA contents.

**Figure 6:**
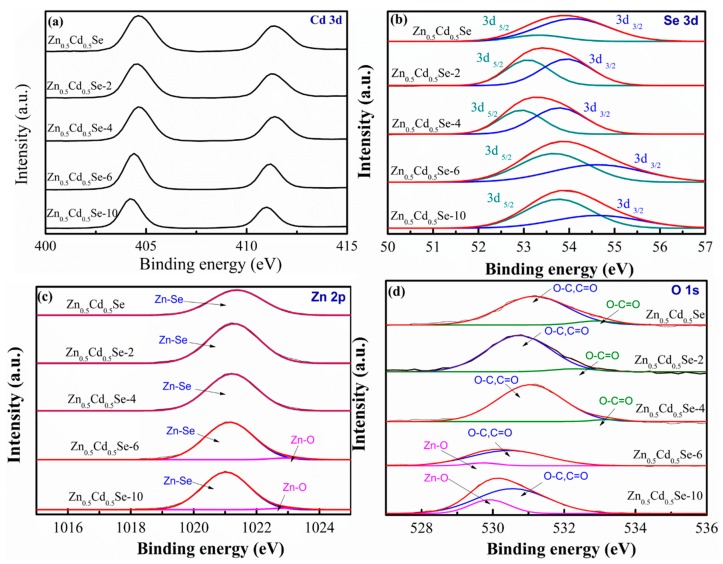
(**a**) Cd 3d, (**b**) Se 3d, (**c**) Zn 2p, and (**d**) O 1s X-ray photoelectron spectroscopy (XPS) spectra showing the influence of the various OLA content on Zn_0.5_Cd_0.5_Se and Zn_0.5_Cd_0.5_Se-y.

To further explain the chemical states and constituent compositions, XPS spectra were analyzed. Figure 6 shows the XPS spectra of Zn_0.5_Cd_0.5_Se and Zn_0.5_Cd_0.5_Se-y samples. In Figure 6a, two peaks (all samples) with binding energies of 404.5 and 411.3 eV can be attributed to Cd 3d [42]. The Se 3d_5/2_ and Se 3d_3/2_ peaks (all samples) with binding energies of 53.7 and 54.6 eV, respectively, are attributed to the Se^2-^ in CdSe and ZnSe, thus confirming the formation of CdSe and ZnSe [42,43], as presented in Figure 6b. In Figure 6c, one peak with binding energy of 1021.2 eV can be observed for Zn_0.5_Cd_0.5_Se, Zn_0.5_Cd_0.5_Se-4, and Zn_0.5_Cd_0.5_Se-6 alloy QDs, which is attributed to Zn^2+^ existing in the form of ZnSe [44,45]. For the Zn_0.5_Cd_0.5_Se-6 and Zn_0.5_Cd_0.5_Se-10 alloy QDs, the two peaks with binding energies of 1021.1 and 1022.7 eV (Figure 6c) can be found, which are assigned to Zn^2+^ in the form of ZnO and ZnSe [44]. As shown in Figure 6d, the XPS spectra can be fit to two peaks with binding energies of 531.2 and 533.1 eV for Zn_0.5_Cd_0.5_Se, Zn_0.5_Cd_0.5_Se-4, and Zn_0.5_Cd_0.5_Se-6 alloy QDs. The binding energy peak at 531.2 eV is attributed to the C–O and C=O bands of oleic acid. The binding energy peak at 533.1 eV is correspondingly attributed to the O–C=O groups of oleic acid [46]. The results indicate that this chemical reaction could not produce ZnO with low OLA contents. For the Zn_0.5_Cd_0.5_Se-6 and Zn_0.5_Cd_0.5_Se-10 alloy QDs (Figure 6d), the O–C=O binding energy peak of oleic acid also appears at 533.1 eV. We also observed that the binding energy peak at 529.9eV is attributed to O^2−^ in ZnO, thus confirming the formation of ZnO [45]. Consequently, the addition of high OLA contents could form ZnO/Zn_0.5_Cd_0.5_Se QDs in this chemical reaction process.

**Figure 7:**
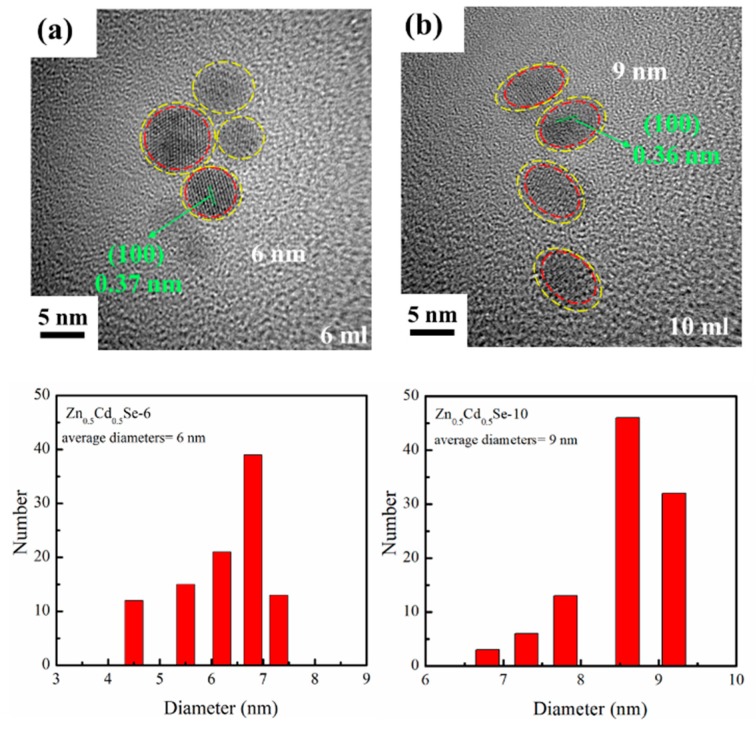
HRTEM images and size distributions showing the influence of the high OLA contents on the (**a**) Zn_0.5_Cd_0.5_Se-6 and (**b**) Zn_0.5_Cd_0.5_Se-10 alloy QDs.

As shown in Figure 7a,b, the HRTEM images and size distributions of Zn_0.5_Cd_0.5_Se-6 and Zn_0.5_Cd_0.5_Se-10 were captured. These figures demonstrate that the Zn_0.5_Cd_0.5_Se-6 and Zn_0.5_Cd_0.5_Se-10 alloy QDs were fully crystalline. Further, they exhibited well-resolved lattice fringes. We clearly observed that adding a high content of OLA during synthesis does not affect the interplanar distances of Zn_0.5_Cd_0.5_Se. In addition, the average diameters were estimated to be approximately 6 and 9 nm for Zn_0.5_Cd_0.5_Se-6 and Zn_0.5_Cd_0.5_Se-10, respectively. The average diameters of the Zn_0.5_Cd_0.5_Se alloy QDs increased as the OLA content increased in the reaction solution from 4 (Figure 2c) to 10 mL. In these images, we found that a thin layer grew on the surface of Zn_0.5_Cd_0.5_Se alloy QDs. This increase might be due to ZnO growing on the Zn_0.5_Cd_0.5_Se surface, thus increasing the particle size.

**Figure 8:**
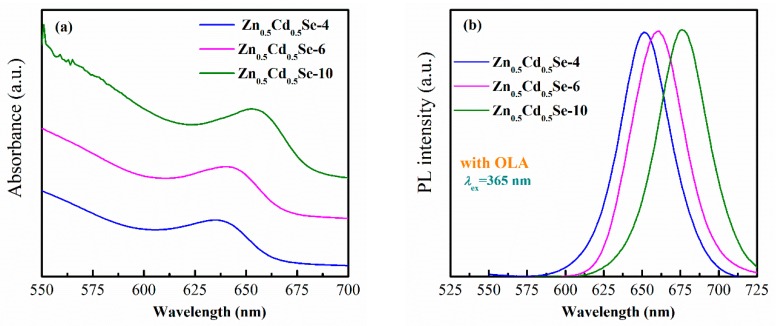
Influence of the high OLA content on the (**a**) UV-vis absorption spectra and (**b**) PL spectra of Zn_0.5_Cd_0.5_Se alloy QDs.

Figure 8 shows the UV-vis absorption and PL spectra of Zn_0.5_Cd_0.5_Se alloy QDs prepared with high OLA contents. After overcoating the Cd_0.5_Zn_0.5_Se with the ZnO shell, the absorption wavelengths of Zn_0.5_Cd_0.5_Se-6 and Zn_0.5_Cd_0.5_Se-10 QDs exhibited a redshift (from 635 to 654 nm) compared to Cd_0.5_Zn_0.5_Se-4 QD, as shown in Figure 8a. Figure 8b shows that the emission peak red-shifted from 651 to 676 nm. The results indicate that ZnO can form in this reaction that grows on the Zn_0.5_Cd_0.5_Se surface due to the increasing reaction between [Zn(OAc)_2_]–OLA complex, TOP, and oxygen ion [47,48,49], thus increasing the particle size and red-shifting the absorption and emission wavelengths. In addition, the photoluminescence quantum yield (PL QY) of all samples with different OLA contents was monitored, calculated by comparison with standard organic dye. The PL QY was estimated to be approximately 21.5%, 14.9%, 13.7%, 23.7%, and 26.5% for pure Zn_0.5_Cd_0.5_Se, Zn_0.5_Cd_0.5_Se-2, Zn_0.5_Cd_0.5_Se-4, Zn_0.5_Cd_0.5_Se-6, and Zn_0.5_Cd_0.5_Se-10, respectively. The PL QY first decreased and then increased with increasing OLA content. This indicates that after Zn_0.5_Cd_0.5_Se cores being coated with ZnO, the PL QY of Zn_0.5_Cd_0.5_Se-6 and Zn_0.5_Cd_0.5_Se-10 obviously enhanced. This phenomenon can be explained by ZnO having a low lattice mismatch with Zn_0.5_Cd_0.5_Se, and ZnO can provide strong confinement for the Zn_0.5_Cd_0.5_Se QD cores as well as remove their surface defects [33,35,36,37,38].

**Figure 9:**
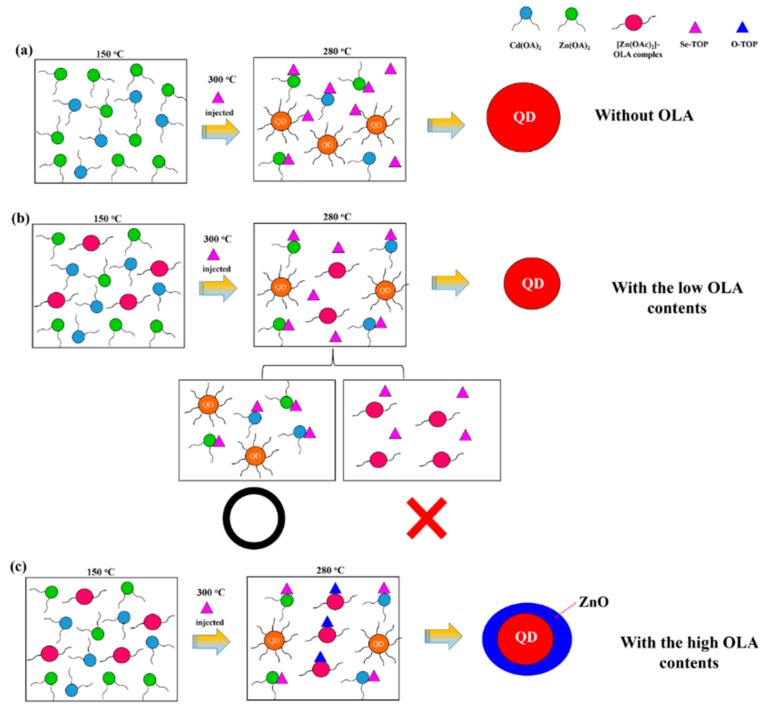
Schematic diagrams of the possible chemical reaction mechanisms underlying the one-pot chemical synthesis of Zn_0.5_Cd_0.5_Se alloy QDs (**a**) without OLA, (**b**) with low OLA contents, and (**c**) with high OLA contents.

To understand all chemical reaction mechanisms, we propose a schematic of the possible reaction mechanism underlying the chemical synthesis for the one-pot method for Zn_0.5_Cd_0.5_Se alloy QDs with low/high OLA contents, as shown in Figure 9. Figure 9a illustrates that the precursors (CdO and Zn(OAc)_2_) in the OA and ODE solution formed Zn(OA)_2_ and Cd(OA)_2_ when the reaction temperature reached 150 °C. When the reaction temperature reached 300 °C, the Se-TOP solution was quickly injected into the reaction solution. At this time, Zn_0.5_Cd_0.5_Se alloy began to form. Subsequently, the reaction temperature was maintained at 280 °C to grow the Zn_0.5_Cd_0.5_Se alloy QDs. This reaction process occurred without the presence of OLA. Figure 9c illustrates the probable chemical synthesis mechanism underlying the growth of Zn_0.5_Cd_0.5_Se alloy QDs in the presence of high OLA contents. It is known that ZnO nanoparticles can be formed through thermal decomposition of Zn(OAc)_2_ or Zn(acac)_2_ [47,48,49]. The literature indicates that before the formation of ZnO nanoparticles, Zn(OAc)_2_ and OLA can form the [Zn(OAc)_2_]–OLA complex as precursors. In this study, when the reaction temperature reached 150 °C, the precursors (CdO and Zn(OAc)_2_) in the OA, OLA, and ODE solution formed Zn(OA)_2_, Cd(OA)_2_, and the [Zn(OAc)_2_]–OLA complex, respectively. Then, cadmium oxide dissociated into cadmium ions and oxygen ions. The [Zn(OAc)_2_]–OLA complex, TOP, and oxygen ion at 280 °C were used to form ZnO via the thermal decomposition with high OLA contents. FTIR, XPS, and XRD data also confirmed that the addition of high OLA contents could form the ZnO/Zn_0.5_Cd_0.5_Se QDs in this chemical reaction process. Thus, the HRTEM results demonstrate that this increase might be due to the ZnO growing on the Zn_0.5_Cd_0.5_Se surface, thus increasing the particle size.

According to the literatures [47,48,49] and above results, we also confirmed that the chemical reaction could produce [Zn(OAc)_2_]–OLA complexes with the addition of low OLA contents (Section 3.1). Because the OLA contents were too low, this chemical reaction could not produce ZnO/Zn_0.5_Cd_0.5_Se QDs (Figure 1 and Figure 5). When the reaction temperature was maintained at 280 °C, only some Zn(OA)_2_ chemically reacted. This phenomenon indicates that the [Zn(OAc)_2_]–OLA complex inhibits the growth of Zn_0.5_Cd_0.5_Se alloy QDs because of the reduction in the reaction between Zn(OA)_2_ and Se^2−^, which leads to a decrease in the particle size, as shown in Figure 9b. Therefore, the Zn_0.5_Cd_0.5_Se alloy QD produces a blue shift of its emission wavelength with increasing the OLA amount (from 0 to 4 mL).

## 4. Conclusions

To the best of our knowledge, a schematic diagram of the possible mechanism for the one-pot synthesis of Zn_0.5_Cd_0.5_Se alloy QDs in the presence of low/high OLA contents is reported for the first time. Under the condition of high OLA contents, the average size of Zn_0.5_Cd_0.5_Se QD examined by HRTEM increases significantly from 4 to 9 nm when the OLA content increases from 4 to 10 mL. In the beginning, the [Zn(OAc)_2_]–OLA complex can be formed via a reaction between Zn(OAc)_2_ and OLA. Then, the thermal decomposition of [Zn(OAc)_2_]–OLA complexes occurs and forms the ZnO as confirmed by the FTIR, XRD, and XPS measurements. The results indicate that the ZnO can grow on the Zn_0.5_Cd_0.5_Se surface, thus increasing the particle size. For the QD synthesized under low OLA loadings of 0, 2, and 4 mL, the average sizes of the Zn_0.5_Cd_0.5_Se alloy QDs are approximately 8, 6, and 4 nm as estimated by HRTEM, respectively. It could be due to the reduction in the reaction between Zn(OA)_2_ and Se^2−^, which led to a decrease in the particle size. Therefore, the emission wavelengths of the Zn_0.5_Cd_0.5_Se alloy QDs are blue-shifted with the increase of the OLA amount from 0 to 4 mL.
